# Role of the nurses in partnering with mothers to give oral medication to their hospitalised child: Modification and development of a contextualised evidence-based practice guideline

**DOI:** 10.4102/curationis.v44i1.2224

**Published:** 2021-09-29

**Authors:** Nadia Harris, Andrea Amos, Natasha North

**Affiliations:** 1 The Harry Crossley Children’s Nursing Development Unit, Department of Paediatrics and Child Health, Faculty of Health Sciences, University of Cape Town, Cape Town, South Africa

**Keywords:** medication, paediatrics, hospital, nursing, safety, guideline, family, mother

## Abstract

**Background:**

In paediatric wards, children are often reluctant to receive medication from nurses and eventually it is given by the parents. It is a common practice for nurses to hand the medication to mothers to give to their children, However, it is an ‘informal’ practice and lacks evidence-based guidelines.

**Objectives:**

To develop a contextualised and adapted evidence-based guideline to support nurses to partner with mothers/carers so that they can safely give oral medication to their hospitalised child under the supervision of a competent nurse.

**Method:**

Existing relevant guidelines were identified through searches of bibliographic databases and websites. The AGREE II: Appraisal of Guidelines for Research and Evaluation II instrument was used to appraise the quality of the identified sources. The process of guideline adaptation recommended by the South African Guidelines Excellence project was followed, and a list of adapted recommendations was developed, aligned with the legislative and regulatory frameworks for nursing in South Africa. Accessible end user documentation was developed.

**Results:**

Six sources were screened and three sources were found to be eligible and were subjected to full appraisal. Two guidelines and one policy document were identified as suitable for adaptation. Expert consultation confirmed that the resulting adapted guideline was sound, easy to understand, and well presented for the target audience.

**Conclusion:**

This process successfully led to the development of a modified evidence-based practice guideline to enable nurses to partner with mothers/caregivers in safely giving oral medication to their hospitalised child in lower-resourced African settings.

## Introduction

Medication errors are one of the most frequent causes of adverse events in the hospital setting and children are believed to be at a higher risk of harm from such errors (Maaskant et al. 2015). Adverse drug events and potential adverse drug events represent a considerable hazard for the paediatric inpatient population; adverse drug events also represent a large cost imposition upon the healthcare sector worldwide (Kunac et al. 2009).

Reports of the incidence of error type in the hospital setting vary. A systematic review of studies related to medication errors in children worldwide found that the most common type of medication error was dosing error (Ghaleb et al. 2006). Other studies have found that the most common medication error by nurses included omissions without valid reason and failure to record (Haw, Stubbs & Dickens 2007). Evidence regarding the extent of the problem in African healthcare settings is scarce (Mekonnen et al. 2018:1–24). A systematic review of publications related to adverse drug events in 18 African hospitals (of which six studies were from South Africa) reported higher rates of medication errors than hospitals from developed countries, although reports were variable and inconsistent (Mekonnen et al. 2018). Reported rates of medication errors in African settings vary considerably, but appear to be higher in paediatric care settings than for adult patients (Mekonnen et al. 2018), possibly because of the added complexity of dosing. Reports of more serious errors appear to be less common, but the consequences of harm can be serious, particularly in situations involving the wrong route of administration, such as an oral suspension given intravenously.

It is the legal responsibility of registered nurses to administer medication to patients, monitor reactions, as well as facilitate the attainment of optimum health for the individual and the family. The execution of the nursing regimen should be according to the legal and regulatory context of South Africa within the scope of practice of the registered nurse (South African Nursing Council [SANC] 2013) and the nurse’s regulation on acts and omissions (South African Nursing Council (SANC) 2014).

Fulfilling these responsibilities is often challenging in the context of lower-resourced African healthcare settings. Factors that contribute to medication errors in African hospitals include environmental factors, such as workplace distraction and high workload, as well as staff fatigue and inadequate knowledge or insufficient training (Mekonnen et al. 2018). Despite the acknowledged scale of the problem, there is limited evidence regarding interventions that are effective in preventing medication errors in the paediatric population (Maaskant et al. 2015) and an almost complete lack of evidence specific to the African settings. It has however been estimated that up to 43.5% of medication errors in African settings may be preventable (Mekonnen et al. 2018). Effective strategies to reduce medication errors in African settings need to be aligned with the context and realities.

A significant issue is the lack of human resources for health in many countries in sub-Saharan Africa (Hoffman et al. 2012). In paediatric wards, medication has to be checked by two registered nurses before it is administered, however this is difficult in low resourced African settings, which often leads to delays in medication administration because a second nurse is not always available on the floor.

A distinctive feature of many African paediatric wards is the presence of a caregiver (often the mother) who is admitted with the child (North et al. 2020). It has been reported that caregivers of admitted children assist with a variety of daily activities including bathing of children, feeding and even monitoring of medication for the children (Hoffman et al. 2012). It is a common practice for nurses to hand the medication to a mother/caregiver to give to their hospitalised child, if a child is reluctant to take the medication from a nurse (Williams, Caldwell & Collins 2016). However, such practice is usually informal and invisible (North et al. 2019). Nurses in lower-resourced settings are often unsure to what degree they ‘should’ involve the mothers and may have concerns about ethics or their scope of practice (Phiri, Kafulafula & Chorwe-Sungani 2017). There is still a gap in the current evidence base for parent participation, especially in developing countries given the higher levels of involvement of mothers/caregivers (Phiri et al. 2017; Power & Frank 2008).

An evidence-based practice approach will assist the nurse in moving away from traditional nursing practices, into a more current research-based approach for better patient care outcomes. Evidence-based nursing practices allow for quality improvement plans, which include clinical practice guidelines, designed to assist with different approaches to better patient care outcomes (Stetler et al. 1998). There is however a lack of high quality evidence-based practice guidelines for nurses partnering with mothers in giving oral medication to their hospitalised children, which are contextually appropriate to a South African setting. The purpose of this study was therefore to develop a contextualised and adapted evidence-based guideline to enable nurses to partner with mothers or caregivers to be competent in giving oral medication to their hospitalised child safely under the supervision of a competent nurse in the South African settings.

## Research methods and design

### Overview of study design

**FIGURE 1:**
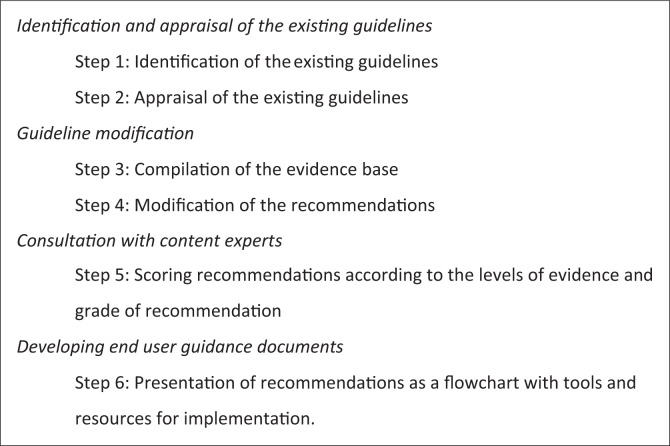
Stages of guideline modification.

A six step method was followed for the modification of guidelines. This incorporated a three-tiered process of guideline modification recommended by Dizon, Machingaidze and Grimmer (2016) as shown in Figure 1.

### Setting

This guideline is intended for use by nurses (registered professional nurse or a child nurse practitioner) and mothers or caregivers partnering together, to safely give oral medication to their hospitalised child in lower-resourced paediatric wards in South Africa.

### Population

A Population–Concept–Context (P-C-C) approach was used to define the focus for the guideline, as follows:

- Population: Children in hospital and their mothers who are involved in their care.
- Concepts: The involvement of mothers in the administration of oral medication to their children.
- Context: Paediatric wards in a lower resourced health facility in Southern Africa.

### Process of guideline development and rationale for guideline modification

In the following sections, a detailed description of the methods used at each of the six stages outlined above (see Figure 1) is provided.

Developing new evidence-based practice guidelines can be time-consuming and expensive, and this can be even more difficult in a low resourced country settings. A more efficient approach could be to adopt, adapt or contextualise recommendations from existing evidence-based guidelines and tailor them to the local context (Dizon et al. 2016).

#### Step 1: Identification of existing guidelines

A structured, transparent and replicable search (PubMed and Cumulative Index to Nursing and Allied Health Literature [CINAHL]), as well as grey literature searches were conducted using keywords to identify the existing guidelines relating to the topic of concern, with a focus on identifying sources relevant to the P-C-C approach described above.

The language selected was English; publication types included journal articles, systematic reviews, guidelines and electronic books. We did not specify a date range as we anticipated that eligible sources would be few and our aim was to be as comprehensive as possible.

#### Step 2: Appraisal of existing guidelines

The AGREE II: Appraisal of Guidelines for Research and Evaluation II instrument was used to appraise the quality of the identified sources (Brouwers et al. 2010).

The AGREE II instrument is a 23-item tool, with six quality domains, which enable assessment of the quality, the appropriate methodological strategies and the rigour of development. Appraisal was carried out independently by three independent reviewers who were all children (or paediatric) nurses and postgraduate students with recent training in the appraisal method. Identified guidelines were appraised individually and the scores were handed back to the project leaders for compilation and resolution of any disparities of more than three. The individual raw scores were summarised, total scores were calculated and then presented in the form of a table.

### Guideline modification

As recommended by Dizon et al. (2016), the process of guideline adaptation should involve compilation of the evidence base prior to reviewing and modifying existing recommendations. A table was created to record the process of modification.

#### Step 3: Compilation of the evidence base

Recommendations of the each included guideline, together with the associated evidence or rationale as stated in the original guideline, were tabulated. The purpose of this was to ensure that any modifications made later would be informed by the relevant evidence base and would preserve the rigour of development of the original source.

#### Step 4: Modification of recommendations

The recommendations were assessed using prompts, which were adapted from the United Kingdom National Institute for Clinical Evidence (NICE) baseline assessment tool (NICE 2015). This prompted consideration of priorities, risks, relevance to the proposed setting and any additional actions that would be needed for implementation in the proposed setting. The table described above was updated to record decisions for each recommendation, which were either to: (1) include without modification, (2) include with modification or (3) exclude with stated reasons.

### Consultation with content experts

Expert input is essential in determining relevance and applicability of research evidence to local contexts (Dizon et al. 2016). Content experts were consulted for expert opinion during the development of the modified guideline as part of a structured series of activities. Consultees included an advanced paediatric nurse practitioner from South Africa familiar with the process of evidence-based guideline development and postgraduate peers at University of Cape Town who were experienced paediatric nursing specialists with training in guideline appraisal and deep experience of local settings and contexts. Consultation centred on eliciting feedback and suggestions for improving draft recommendations and end user documentation, related to prioritisation and relevance, risk and safety implication issues, and comprehensive application of the evidence base.

#### Step 5: Scoring recommendations according to levels of evidence and grade of recommendation

For each of the new or adapted practice recommendations, we assessed whether there was a need to provide a supporting rationale or whether the existing rationale needed to be strengthened or contextualised with evidence specific to the setting. We undertook further focused literature searches with guidance from a specialist librarian to ensure that all recommendations were supported by evidence-based rationale statements. We assigned each recommendation and rationale a score for the level of evidence from I (highest quality) to V (least robust), and the grade of recommendation from A (strong evidence of efficacy, strongly recommended) to E (strong evidence against efficacy or for adverse outcome, never recommended) using the approach described by Xynos et al. (2016).

An additional scoring system was developed to support recommendations that related to the legislative and regulatory frameworks that govern nursing practices in South Africa and which could be regarded as mandatory recommendations. These recommendations were indicated as a legal requirement (LR) or regulatory requirement (RR).

### Developing end user guidance documents

We wanted to ensure that the key messages of our guideline would be accessible to nurses. A flowchart is a ‘process map’ with easy to understand diagrams of step-by-step instructions to assist in understanding an existing process or develop ideas about how to improve it (Institute for Health Improvement 2020). Flowcharts are valuable in nursing as they allow for an accurate step-by-step process of how to perform a specific task, which allows for quick and efficient patient care that is in line with the policy and the protocol. Flowcharts can help nurses make quick and safe decisions to prioritise actions (Sánchez et al. 2007). As the final step in the process of guideline modification, we created a flowchart as a visual representation of the adapted and contextualised recommendations.

## Results

The six step methodology described above resulted in the following outcomes at each step.

### Step 1: Guideline identification

The bibliographic database search, grey literature search, and the consultation resulted in the identification of six sources. After initial screening by title and summary, two guidelines and one policy statement were selected for full text review as they met the inclusion criteria for our search. The guidelines were as follows:

- Guideline 1: ‘Guy’s and St. Thomas’ – National Health Service (NHS) (UK) Foundation Trust. (2018). Clinical Guideline/Protocol for self/carer administration of medicines to adults and paediatric patients’.
- Guideline 2 (policy statement): ‘Aberdeenshire Council (2016). Supporting Children and Young People with Health Care Needs and Managing Medicines in Educational Establishments. NHS Grampian – Aberdeenshire Council’.
- Guideline 3: ‘NICE guideline [NG5] Published date: (2015). Medicines optimisation: The safe and effective use of medicines to enable the best possible outcomes’.

### Step 2: Appraisal of the existing guidelines

The individual raw scores were summarised and total scores were calculated as shown in Table 1. The widest range of scoring was found in Domain 3 (rigour of development). This may reflect the availability of information about the process of development, which was not fully described in all cases. Guideline authors were contacted by email to request additional information, however feedback received did not include any additional information.

**TABLE 1:** Domain scores obtained following guideline appraisal using the AGREE II tool.

Publication details	Domain 1: Scope and purpose (%)	Domain 2: Stakeholder involvement (%)	Domain 3: Rigour of development (%)	Domain 4: Clarity of presentation (%)	Domain 5: Applicability (%)	Domain 6: Editorial independence (%)	Overall guideline assessments by reviewer				Recommend guideline: Yes OR Yes, with modification OR No
							1	2	3	4	
**Title:** Medications optimisation: The safe and effective use of medicines to enable best possible outcomes.	89	85	86	72	58	65	6	6	6	6	Yes, With modification
**Institution:** National Institute for Health and Care Excellence											
**Year:** 2015											
**Country:** England.											
**Title:** Protocol for self/carer administration of medicines to adult and paediatric patients.	50	46	13	81	39	3	5	4	6		Yes, with modification
**Institution:** Guy’s and St Thomas’ NHS Foundation Trust.											
**Author:** Barnes											
**Year:** 2018											
**Country:** England.											
**Title:** Supporting children and young people with healthcare needs and managing medicines in educational establishments	78	61	28	25	44	0	3	3			Yes, with modification
**Institution:** NHS Grampian-Aberdeenshire Council											
**Institution:** Aberdeenshire Council											
**Year:** 2016											
**Country:** Scotland											

NHS, National Health Service.

## Guideline adaptation

### Step 3: Compilation of the evidence base

Levels of evidence for the relevant recommendations in the existing guidelines were scored between II (small or large randomised control trials (RCTs) with a suspicion of bias, or meta-analysis of such trials with demonstrated heterogeneity) and V (studies without control group, case reports and expert opinion).

As a result of this exercise, informed by the available evidence about medication errors in African settings, we decided that the priority areas for our guideline to focus on should be:

- Supporting nurses to partner with mothers to give medication to their child, working within a well-defined professional and ethico-legal framework
- Actions to prevent the most common and the most serious types of reported medication errors: omissions without valid reason and failure to record and wrong route of administration.

### Step 4: Modification of recommendations

A total of 40 recommendations relevant to the topic were identified from the included sources. After consideration of priorities, risks and relevance to the proposed setting, none of the recommendations were identified as suitable to include without modification. A total of 21 recommendations were excluded for stated reasons including lack of applicability to the settings. A total of 19 recommendations were identified as suitable to include with modification. This remaining set of 19 recommendations contained closely similar material and the list was edited and further reduced to a final list of 12 adapted and contextualised recommendations, which were categorised into four themes (assessment, teaching, implementation and documentation) aligned with the nursing process (with the help of expert consultation). These are presented in Table 2.

**TABLE 2:** Recommendations with level of evidence and grade of recommendation.

Recommendation	Rationale	References	Level of evidence	Grade of recommendation
**1. Assessment: Assessing eligibility to enrol the mother/carer in the administration of oral medication**				
1.1 Mothers/carers participating in administration of oral medication for their child should be assessed by a competent nurse to ensure that they meet the inclusion and exclusion requirements using Tool 1 (Assessment of mothers’ understanding and willingness to participate)	Assessment of the mother/carer’s capabilities is in the best interest of the patient’s safety with regard to giving medication. A record of the assessment acts as proof that the mother willingly participates and was found competent by the attending nurse.	Guy’s and St. Thomas’ - NHS Foundation Trust (2018)	Level 5/**LR/RR**	Grade C/**LR/RR**
	According to R786, (Act No. 33 of 2005) No. 4.1, the professional nurse must be competent and responsible for managing a patient’s condition and providing comprehensive nursing care treatment and ensuring safe implementation of nursing care and the delegation of nursing care to competent practitioners or persons.	SANC R786 (Act No. 33 of 2005) No. 4.1		
1.2 The nurse should obtain informed consent from the mother/carer prior to enrolling them in giving oral medication to their hospitalised child.	Informed consent that has been recorded acts as proof of the mother/carer’s willingness to participate, and that she understood the information given to her to make an informed decision. It also indicates that the nurse has explained and given necessary information regarding the administration of oral medications and the responsibilities that lie with the nurse and mother.	Guy’s and St. Thomas’- NHS Foundation Trust (2018)	Level 5/**LR/RR**	Grade C/**LR/RR**
	Scope of practice a registered nurse entails are: treatment and administration of medication to patients and monitoring of reactions, as well as the facilitation of attainment of optimum health for the individual and family in execution of the nursing regimen.	Scope of practice of the Registered nurse R2598 (No. 50 of 1978) Chapter 2- 2(C) & (O)		
	The responsibility of the registered nurse is to assess the healthcare information needs, plans and respond accordingly.	SANC Revised Version: R786, (Act No. 33 of 2005) No. 4.3 (i)		
	Providing accurate and truthful information relating to informed consent or refusal to enable the individual to make an informed decision.	SANC 2013, Code of Ethics, (No. 4- Value Statement)		
1.3 When discussing administration of oral medication with the mother/carer, the nurse should consider the following: The mother’s/carer’s knowledge and skills needed to be able to give oral medication to her child in hospital (use Tool 1 and Tool 2). The benefits and risks of mother/carer administration of oral medication. The mother’s/carers values, preferences and willingness to give oral medication in the ward. Any support or monitoring the mother/carer needs.	By ensuring the mother/carer is adequately equipped to carry out the task of giving oral medication to the child whilst in hospital, they will also be prepared to carry on giving oral medication after discharge (if necessary). Implementation of this recommendation can identify and eliminate potential risk factors. The mother or carer will feel supported during her role of giving oral medication to her child, as well as having their preferences considered.	NICE guideline 5 guidance.nice.org.uk/NG5 (2015)	Level 2	Grade B
	Self-management of medications is safe, reliable and effective, for a sizable proportion of patients.	Fitzmaurice et al. (2005)		
	Grunau, Wiens and Harder (2011) showed that although self-management plans are not more effective or more ‘superior’ to primary healthcare plans, self-management plans were preferred by patients.	Grunau et al. (2011)		
	Thoonen et al. (2003) found that patients with asthma perceived that self-management plans lowered the burden of diseases with asthma and concluded that self-management plans are at least as safe and effective as treatment plans in primary care.	Thoonen et al. (2003)		
1.4 On completion of the assessment, some mothers/carers will not be considered competent or will not agree to participate with oral administration of medication to the child. In these instances, the nurse will retain the complete responsibility for the administration of medicines.	The overall responsibility for giving medication in the ward belongs to the nurse, who is accountable for treatment being given and any adverse events. Mothers are not obligated to perform nursing duties. Having the mother at the bedside is both beneficial to the nurse, the mother, and the child. However, mothers must not be forced to give oral medication to children and must not be expected to perform such duties if they are not feeling well or do not wish to participate in this activity.	Guy’s and St. Thomas’- NHS Foundation Trust (2018)	Level 5/**LR/RR**	Grade C/**LR/RR**
	According to North et al. (2020): ‘the presence of mothers can support the smooth running of the ward, reducing demands on nurses and contributing to faster healing and recovery for the child’. Effective nurse–mother partnering requires nurses to trust mothers to be responsible for aspects of their child’s care, whilst nurses continue to supervise some aspects of care.	North et al. (2020)		
	A professional nurse must be competent and is responsible for ensuring safe implementation of nursing care and the delegation of nursing care to competent practitioners or persons. This recommendation is vital in ensuring patient safety and completion of tasks for which the nurse is ultimately accountable.	SANC R786 (Act No. 33 of 2005) No. 41		
**2. Teaching**				
2.1 In order to give oral medication to the hospitalised child safely, mothers/carers should be educated and assessed by a competent nurse and at a minimum be able to: Recognise the medication (by name or appearance). Know when to give it. Understand the correct dose. Have some appreciation of its purpose. Recognise any signs of adverse or allergic reactions to treatment.	The nurse needs to assess the mother/carer’s suitability or competency prior to them assisting with giving of oral medication to their children. If the mother/carer is unable to identify a medication, understand its purpose or any allergic reactions, it may lead to serious adverse events, which could become fatal to the child.	Guy’s and St. Thomas’- NHS Foundation Trust (2018)	Level 2	Grade B
	Patient education programmes can improve self-management and/or outcomes.	Grunau et al. (2011)		
	Patient education can be as important as proper recording by patients in diaries for effective use and outcomes of self-management plans.	Thoonen et al. (2003)		
2.2 Nurses are responsible for explaining to the mother/carer and child where appropriate, how to identify and report patient safety incidents and any error or incident in the administration of oral medicine to a child which is observed or discovered by a nurse or mother/carer, must be reported to the nursing manager and the attending doctor, and an incident report must then be completed by the nurse.	The nurse is responsible and accountable for patient safety in the ward. The nurse needs to teach the mother/carer how to report safety incidents to reduce any complications and to find ways of preventing them from happening in the future. Mothers or carers are valuable assets in the unit and can be very helpful in ensuring better healthcare outcomes for children.	NICE guideline 5 guidance.nice.org.uk/NG5 (2015)	Level 4**/LR/RR**	Grade B/**LR/RR**
	North et al. (2020) discussed high-quality African nursing practices where mothers were expected by nurses to become quickly competent at managing the hospitalised child’s health needs and participate in care such as observing the child’s condition and reporting changes and concerns.	North et al. (2020)		
	Medication errors are common in the wards.	Haw et al. (2007)		
	Kunac et al. (2009) found that adverse drug events and potential adverse drug events represent a considerable hazard for the paediatric inpatient population and adverse drug events in addition represent a large cost imposition upon the healthcare sector.	Kunac et al. (2009)		
	The accountability for medication administration lies with the registered nurse in the ward. The nurse has to ensure patient safety measures. If there are any medication errors or adverse events it is the duty of the nurse to report these events and ensure measures are taken to reduce further harm to the patient. If not implemented correctly, it could have serious implications on patient safety and quality of care. Recommendation was adapted and then added on to suit the context of the nurse, mother/carer and child. The registered nurse is accountable for ensuring correct treatment and medication for patient care and all records are correct.	Guy’s and St. Thomas’- NHS Foundation Trust (2018)		
	The registered nurse is accountable for any acts or omissions with regard to patient care.	SANC (2014)		
	All errors must be recorded and reported to the patient’s doctor and the superintendent of the healthcare facility. Above all, the nurse must identify, correct and report errors.	Human and Mogotlane (2017)		
2.3 If changes are made to medicines, additional support and necessary education must be provided to the mother/carer, as required and mother or carer to be assessed as competent prior to giving new medication to her child.	In order for the mother to appropriately and correctly give oral medication to the child, she should be informed, supported and educated when changes are made to the child’s medication, in order to continue giving the child’s medication safely.	Guy’s and St. Thomas’- NHS Foundation Trust (2018)	Level 5	Grade C
**3. Implementation**				
3.1 There may be times when the mother or carer will be temporarily unable to participate in oral administration of medication to hospitalised children. For example, Nil by Mouth, immediate pre/post-operative period, following sedation. Following an explanation to the mother or carer, the nurses should resume sole responsibility for oral administration of medication to the child.	Patient’s conditions are likely to change whilst in hospital. The mother cannot be expected to share the responsibility for administration of medication in children who are unstable. The recommendation was adapted to suit the context of the nurse, mother or carer and child.	Guy’s and St. Thomas’- NHS Foundation Trust (2018)	Level 5/**RR/ LR**	Grade C/**LR/RR**
	Scope of practice a registered nurse entails include treatment and administration of medication to patients and monitoring of reactions, as well as the facilitation of attainment of optimum health for the individual and family in execution of the nursing regimen.	SANC Scope of practice of the Registered nurse R2598		
	The responsibility of the registered nurse is to assess the healthcare information needs, plans and respond accordingly.	SANC R786 (Act No. 33 of 2005)		
3.2 The nurse is responsible for handing over prescribed doses to the mother or carer during medication rounds.	The registered nurse is accountable for the safe administration of medication in the wards, as well as giving the prescribed doses. It is not the responsibility of the mother to store medication and calculate prescribed doses.	Aberdeenshire Council (2016)	Level 5/**RR/LR**	Grade C/**RR/LR**
	The registered nurse is responsible for the treatment, care and the administration of medicine to a patient.	SANC Scope of practice of the Registered nurse R2598 (No. 50 of 1978)		
	The nurse is responsible for the administration of medication, once she has accepted a prescription. The nurse must be sure to supervise treatment she does not administer personally, to ensure that it is given correctly by those under her supervision.	Human and Mogotlane (2017)		
3.3 The nurse must continue to observe the patient for any signs of adverse reactions and monitor the effectiveness of medication.	Nurses are trained to recognise deterioration in a patient’s condition and assessing whether a patient’s prognosis is improving. The overall responsibility for the patient still belongs to the nurse, which means patient safety is a priority for the nurse.	Guy’s and St. Thomas’- NHS Foundation Trust (2018)	Level 5/**RR/LR**	Grade C/**RR/LR**
	Scope of practice of a registered nurse entails treatment and administration of medication to patients and monitoring of reactions.	Scope of practice of the Registered nurse R2598 No. 50		
	Registered healthcare professionals including nurses who are accountable when handling medications and need to be competent, legally entitled and trained, authorised to do their jobs.	Royal Pharmaceutical Society of Great Britain, 2020, Professional guidance on the safe and secure handling of medicines		
	The nurse must continuously observe the patient’s physical and psychological state and take action to prevent regression of the patient’s condition.	Human and Mogotlane (2017)		
**4. Documentation**				
4.1. The nurse should ensure that the mother/carer completes a medication record using the tick sheet-record tool (Tool 3) provided each time they give medication to their child and the nurse must also record administration on the inpatient chart.	Signing for medication given in partnership by both the nurse and mother or carer, acts as proof that the medication has been administered. During handover of the patient, the nurse taking over will be aware that the mother has given the medication and the nurse that handed over had supervised and made sure that the mother had given the child’s oral medication.	Guy’s and St. Thomas’- NHS Foundation Trust (2018) Aberdeenshire Council (2016)	Level 5/**LR/RR**	Grade C/**LR/RR**
	The registered nurse is responsible for the recording of data on assessment and intervention outcomes and documenting information in a meaningful manner for improving quality of care.	South African Nursing Council (2004), Charter of Nursing Practice Draft 1		
	An authorised nurse who supplies, administers and prescribes a medicine listed in schedule 1–6 of the medicines Act is responsible for recording of medication being given to patients.	Government Gazette, 2009, Medicines and Related Substances Amendment Act, 72 of 2008, South African Government		
4.2 The nurse should review the mother/carer oral administration plan every 3 days or when changes occur to ensure the mother/carer does not have any problems using it, for example, psychosocial changes, physical illness or feeling incompetent etc.	The nurse is not only responsible for the welfare of the child but the welfare of the mother as well. The nurse is to ensure that the mother is still comfortable with performing tasks and ensuring the mother/carer feels valued and her needs and challenges are acknowledged and tended to. If the mother/carer feels the tasks are too much for her or she has ideas on ways to make things better to suit her, not only in hospital but after discharge as well, it is vital that her voice is heard. If this recommendation is not implemented correctly, it may affect relationships between nurse and mother/carer.	NICE guideline 5 guidance.nice.org.uk/NG5 (2015)	Level 2	Grade B
	Individualised home-management plans (with written guidelines) have been found to improve and control persistent asthma.	Agrawal et al. (2007)		
	A randomised crossover comparison study on self-management plans and normal conventional institutionalised care plans, revealed more patient satisfaction from those who were enrolled in the self-management programme.	Cromheecke et al. (2000)		
	A randomised crossover trial was conducted, assessing the efficacy of patient self-management of anticoagulation with warfarin. The study found that most of the participants preferred self-management plans.	Grunau et al. (2011)		

RR, Regulatory requirement; LR, Legal Requirement; SANC, South African Nursing Council; NICE, National Institute for Clinical Evidence; NHS, National Health Service; NG, NICE guidelines.

### Step 5: Scoring new recommendations according to levels of evidence and grade of recommendation

For the final list of 12 recommendations, the levels for the grades of evidence scored between II and V and strength of recommendations were graded between B and C, which was the same as before modification. Recommendations graded C were additionally supported by feedback from the clinical experts consulted, with the low grading thought to reflect a lack of impact studies rather than a likelihood of ineffectiveness.

Expert consultation confirmed that the resulting adapted guideline was sound, easy to understand and well-presented for the target audience.

### Developing end user guidance documents

#### Step 6: Presentation of recommendations

**FIGURE 2:**
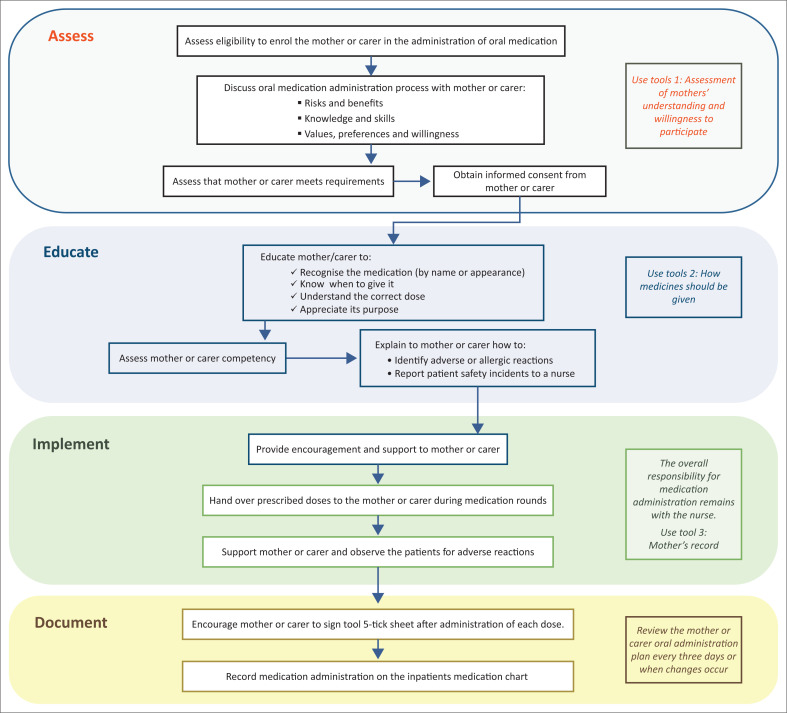
Flowchart summary to guide nursing actions.

A flowchart (Figure 2) was developed to present the 12 final recommendations as an easy way to understand visual representation of the recommendations in the form of step-by-step instructions, grouped into four themes as a quick reference for nurses in the ward. Supporting tools were included for the enrolment and training of the mother or caregiver, adapted from the Guy’s and St. Thomas’ guideline (2018). End user documentation has been made available online (see data availability statement), which include: assessment of mothers’ understanding and willingness to participate (Tool 1); and how the medicines should be given (Tool 2).

## Discussion

### Key findings

It has been acknowledged that current practice in many wards is for nurses to hand medication to mothers to give to their children, but this is usually an ‘informal’ practice and lacks evidence-based guidelines. We found that this issue is amenable to improvement through evidence-based guidance (Williams et al. 2016), and that there was a need for a guideline specific to the population and setting. Our contextualised and adapted evidence-based guideline is intended to enable nurses to partner with mothers or carers (regardless of literacy levels) to be competent and safely give oral medication to their hospitalised child under the supervision of a competent nurse in South African settings.

### Discussion of key findings

We found limited evidence regarding interventions that are effective in preventing medication errors in the paediatric population (Maaskant et al. 2015), and no examples of high-quality evidence-based guidelines specific to nurses partnering with mothers to give medication in paediatric settings. However, the strength of evidence regarding the positive impact of interventions that promote self-care and education about medications management is good (West-Oram, Lister & Dougherty 2015), suggesting that the recommendations are likely to be beneficial.

We identified two main categories of problem as the focus for our contextualised guideline. Firstly, we prioritised the need for the guideline to support nursing practice by aligning with a well-defined professional and ethico-legal framework. Nurses working without explicit policies concerning partnering with mothers find that practice varies between wards and sisters in charge, leaving nurses unsure of what is an acceptable practice and how to fulfil their professional responsibilities (Phiri et al. 2017). Involving mothers in giving medication seems to be an invisible and informal nursing practice (North et al. 2019). Informal nursing practices are difficult to reflect on or improve, so an important first step is to recognise and acknowledge the practice of nurses partnering with mothers to give medication to their hospitalised children. Once this is recognised, nurses will be better able to reflect on their practices and take any necessary steps to improve them. Even when informal practices are acknowledged, nurses may be unsure of the extent to which they can delegate (Human & Mogotlane 2017). In our experience, nurses are often unclear about whether they can delegate responsibility for giving medication to a child’s mother and may wrongly assume they should not be doing this. By addressing the scope of practice and clarifying how a competent nurse can responsibly delegate this activity, in accordance with the regulations and after obtaining informed consent, we have tried to ensure our guideline will be understandable and acceptable to nurses, and that they provide a basis for informed professional practice. We have also included an assessment of mothers’ competence as a practical tool to support nurses in responsibly delegating to mothers (after obtaining informed consent) whilst remaining in a supervisory role.

Secondly, the area that we prioritised was to focus on nursing actions to prevent the most common and the most serious reported types of medication errors (Maaskant et al. 2015): omissions without valid reason and failure to record and wrong route of administration. As medication errors in children are one of the most frequent causes of adverse events in the hospital setting, we hoped to address barriers to improving practice in order to support implementation. The recommendations are intended to reduce the workload for nurses, by safely enrolling mothers in active care giving. This active involvement can have other benefits.

Involvement of mothers in the care of the child forges relationships between the mother and the nurse, and encourages the family of the child to have a sense of trust in the nurses working closely with the child and the mother (Salmani, Abbaszadeh & Rassouli 2014). High-quality African nursing practices have been described, where mothers were expected by nurses to become quickly competent at managing the hospitalised child’s health needs, and participate in care such as observing the child’s condition, tube feeding, prescribed physiotherapy exercises, providing a reassuring presence for the child during procedures and dressing changes and assisting with giving medication (North et al. 2020). This suggests that it is feasible to involve mothers in this aspect of care-giving and that nurses can do so with confidence, supported by evidence, as part of strategies to reduce medication errors and improve safety for children in hospital.

### Strengths and weaknesses

The focus of our guideline is on supporting nurses to partner with mothers to give medication. We have assumed that registered professional nurses will be competent to fulfil this role. Additional work to support nurses in maintaining and updating their knowledge in respect of medicines management would be valuable.

Although we ensured that all recommendations were supported by evidence-based rationale statements, we were limited by the availability of evidence. It was beyond the scope of our study to undertake a systematic review for every recommendation. Instead we have transparently presented the results from limited but focused literature searches. We recommend further research to build the evidence base on this topic, particularly looking at the outcomes of interventions.

### Recommendations

Prior to implementing this guideline, the nurse with overall responsibility for medicines management on the ward should agree to a list of medications with the head pharmacist, the head clinician and the nursing unit manager on which medications can and cannot be handed to mothers for administration to their children. The overall responsibility for administration of oral medication in the ward belongs to the nurse. The nurse will have continuing responsibility for recognising and acting upon changes in a patient’s condition, regardless of the mother/carer’s participation in the process (Guy’s and St. Thomas’- NHS Foundation Trust 2018).

## Conclusion

This process successfully led to the development of a modified evidence-based practice guideline to enable nurses to partner with mothers or carers in safely giving oral medication to their hospitalised child in South African settings. The guideline can be used by nurses to review and improve the care offered to children, with the aim of contributing to a safer care through reduced medication errors.

## References

[CIT0001] Aberdeenshire Council, 2016, ‘Supporting children and young people with health care needs and managing medicines in educational establishments’, *NHS Grampian, Aberdeenshire Council: Scotland*, viewed 29 January 2020, from https://asn-aberdeenshire.org/wp-content/uploads/2021/03/Supporting-Children-

[CIT0002] Agrawal, S., Singh, M., Mathew, J. & Malhi, P., 2007, ‘Efficacy of an individualized written home-management plan in the control of moderate persistent asthma: A randomized, controlled trial’, *Acta Paediatrica* 94(12), 1742–1746. 10.1080/08035250510039973 16421033

[CIT0003] Brouwers, M.C., Kho, M.E., Browman, G.P., Burgers, J.S., Cluzeau, F., Feder, G. et al., 2010, ‘AGREE II: Advancing guideline development, reporting and evaluation in health care’, *CMAJ* 182(18), E839–E842. 10.1503/cmaj.090449 20603348 PMC3001530

[CIT0004] Cromheecke, M., Levi, M., Colly, L., De Mol, B., Prins, M., Hutten, B.A. et al., 2000, ‘Oral anticoagulation self-management and management by a specialist anticoagulation clinic: A randomised cross-over comparison’, *The Lancet* 356(9224), 97–102. 10.1016/S0140-6736(00)02470-3 10963245

[CIT0005] Dizon, J.M., Machingaidze, S. & Grimmer, K., 2016, ‘To adopt, to adapt, or to contextualise? The big question in clinical practice guideline development’, *BMC Research Notes* 9, 442. 10.1186/s13104-016-2244-7 27623764 PMC5022236

[CIT0006] Ghaleb, M.A., Barber, N., Franklin, B.D., Yeung, V.W., Khaki, Z.F. & Wong, I.C., 2006, ‘Systematic review of medication errors in pediatric patients’, *Annals of Pharmacotherapy* 40(10), 1766–1776. 10.1345/aph.1G717 16985096

[CIT0007] Grunau, B.E., Wiens, M.O. & Harder, K.K., 2011, ‘Patient self-management of warfarin therapy: Pragmatic feasibility study in Canadian primary care’, *Canadian Family Physician Medecin de Famille Canadien* 57(8), e292–298, viewed 22 April 2020, from https://europepmc.org/article/med/21841092. 21841092 PMC3155464

[CIT0008] Guy’s and St. Thomas’- NHS Foundation Trust, 2018, ‘Clinical guideline/protocol for self/carer administration of medicines to adults and paediatric patients’, Unpublished.

[CIT0009] Haw, C., Stubbs, J. & Dickens, G., 2007, ‘An observational study of medication administration errors in old-age psychiatric inpatients’, *International Journal for Quality in Health Care* 19(4), 210–216. 10.1093/intqhc/mzm019 17562662

[CIT0010] Hoffman, M., Mofolo, I., Salima, C., Hoffman, I., Zadrozny, S., Martinson, F. et al., 2012, ‘Utilization of family members to provide hospital care in Malawi: The role of hospital guardians’, *Malawi Medical Journal* 24(4), 74–78, viewed 21 April 2020, from https://www.ajol.info/index.php/mmj/article/view/85665. 23638281 PMC3623026

[CIT0011] Human, S. & Mogotlane, S., 2017, *Professional practice: A South African nursing perspective*, 6th edn., Pearson: Cape Town, South Africa.

[CIT0012] Institute for Health Improvement, 2020, *Flow chart*, Cambridge, MA, viewed 29 June 2020, from http://www.ihi.org/resources/Pages/Tools/Flowchart.aspx.

[CIT0013] Kunac, D., Kennedy, J., Austin, N. & Reith, D., 2009, ‘Incidence, preventability, and impact of adverse drug events (ADEs) and potential ADEs in hospitalized children in New Zealand’, *Pediatric Drugs* 11(2), 153–160. 10.2165/00148581-200911020-00005 19301935

[CIT0014] Maaskant, J., Vermeulen, H., Apampa, B., Fernando, B., Ghaleb, M., Neubert, A. et al., 2015, ‘Interventions for reducing medication errors in children in hospital’, *Cochrane Database of Systematic Reviews* 10(3), CD006208, viewed 27 January 2020, from https://pubmed.ncbi.nlm.nih.gov/25756542/. 10.1002/14651858.CD006208.pub3 PMC10799669 25756542

[CIT0015] Mekonnen, A.B., Alhawassi, T.M., McLachlan, A.J. & Brien, J.E., 2018, ‘Adverse drug events & medication errors in African hospitals: A systematic review’, *Drugs-real World Outcomes* 5(1), 1–24. 10.1007/s40801-017-0125-6 29138993 PMC5825388

[CIT0016] NICE, 2015, *Baseline assessment tool for medicines optimisation*, NICE Medicines Practice Guideline NG5, viewed 30 March 2020, from https://www.nice.org.uk/about/what-we-do/into-practice/audit-and-service-improvement/assessment-tools.

[CIT0017] NICE guideline [NG5], 2015, *Medicines optimisation: The safe and effective use of medicines to enable the best possible outcomes*, viewed 19 February 2020, from https://www.nice.org.uk/guidance/ng5. 26180890

[CIT0018] North, N., Leonard, A., Bonaconsa, C., Duma, T. & Coetzee, M., 2020, ‘Distinctive nursing practices in working with mothers to care for hospitalised children at a district hospital in KwaZulu-Natal, South Africa: A descriptive observational study’, *BMC Nursing* 19(1), 28. 10.1186/s12912-020-00421-1 32327935 PMC7169043

[CIT0019] North, N., Sieberhagen, S., Leonard, A., Bonaconsa, C. & Coetzee, M., 2019, ‘Making children’s nursing practices visible: Using visual and participatory techniques to describe family involvement in the care of hospitalized children in Southern African settings’, *International Journal of Qualitative Methods* 18, 1–14. 10.1177/1609406919849324

[CIT0020] Phiri, P.G., Kafulafula, U. & Chorwe-Sungani, G., 2017, ‘Registered nurses’ experiences pertaining to family involvement in the care of hospitalised children at a tertiary government hospital in Malawi’, *Africa Journal of Nursing and Midwifery* 19(1), 131–143. 10.25159/2520-5293/910

[CIT0021] Power, N. & Franck, L., 2008, ‘Parent participation in the care of hospitalized children: A systematic review’, *Journal of Advanced Nursing* 62(6), 622–641. 10.1111/j.1365-2648.2008.04643.x 18503645

[CIT0022] Salmani, N., Abbaszadeh, A. & Rassouli, M., 2014, ‘Factors creating trust in hospitalized children’s mothers towards nurses’, *Iranian Journal of Pediatrics* 24(6), 729–738, viewed 06 November 2020, from https://pubmed.ncbi.nlm.nih.gov/26019779/. 26019779 PMC4442835

[CIT0023] Sánchez, M., López, B., Bragulat, E., Gómez-Angelats, E., Jiménez, S., Ortega, M. et al., 2007, ‘Triage flowchart to rule out acute coronary syndrome’, *The American Journal of Emergency Medicine* 25(8), 865–872. 10.1016/j.ajem.2006.12.025 17920969

[CIT0024] South African Nursing Council (SANC), 2004, *Charter of nursing practice draft*, viewed 23 April 2020, from https://pdf4pro.com/view/draft-charter-of-nursing-practice-sa-nursing-1902.html

[CIT0025] South African Nursing Council (SANC), 2013, *Code of ethics for nursing*, viewed 24 April 2020, from https://www.sanc.co.za/wp-content/uploads/2021/04/Code-of-Ethics-for-Nursing-in-South-Africa.pdf

[CIT0026] South African Nursing Council (SANC), 2013, *R786: Regulations regarding the scope of practice of nurses and midwives*, viewed 09 September 2021, from https://www.sanc.co.za/wp-content/uploads/2020/07/gg38095_nn786_Midwifery.pdf

[CIT0027] South African Nursing Council (SANC), 2014, *R767: Regulations setting out the acts or omissions in respect of which the council may take disciplinary steps*, viewed 09 September 2021, from https://www.sanc.co.za/wp-content/uploads/2020/06/R767-Reg-act-2014.pdf

[CIT0028] Stetler, C.B., Brunell, M., Giuliano, K.K., Morsi, D., Prince, L. & Newell-Stokes, V., 1998, ‘Evidence-based practice and the role of nursing leadership’, *JONA: The Journal of Nursing Administration* 28(7/8), 45–53. 10.1097/00005110-199807000-00011 9709696

[CIT0029] Thoonen, B., Schermer, T., Van den Boom, G., Molema, J., Folgering, H., Akkermans, R. et al., 2003, ‘Self-management of asthma in general practice, asthma control and quality of life: A randomised controlled trial’, *Thorax* 58(1), 30–36. 10.1136/thorax.58.1.30 12511716 PMC1746452

[CIT0030] West-Oram, A., Lister, S.E. & Dougherty, L., 2015, ‘The Royal Marsden manual of clinical nursing procedures, student edition’, 9th edn., viewed 10 March 2020, from https://www.academia.edu/42155253

[CIT0031] Williams, L., Caldwell, N. & Collins, E., 2016, ‘Helping parents/carers to give medicines to children in hospital’, *Archives of Disease in Childhood* 101(9), e2, 10.1136/archdischild-2016-311535.13 27540191

[CIT0032] Xynos, E., Gouvas, N., Triantopoulou, C., Tekkis, P., Vini, L., Tzardi, M. et al., 2016, ‘Clinical practice guidelines for the surgical management of colon cancer: A consensus statement of the Hellenic and Cypriot colorectal cancer study group by the HeSMO’, *Annals of Gastroenterology* 29(1), 3–17, viewed 10 April 2020, from https://www.ncbi.nlm.nih.gov/pmc/articles/PMC4700843/pdf/AnnGastroenterol-29-3.pdf. 26752945 PMC4700843

